# Melatonin as a Novel Interventional Candidate for Fragile X Syndrome with Autism Spectrum Disorder in Humans

**DOI:** 10.3390/ijms18061314

**Published:** 2017-06-20

**Authors:** Jinyoung Won, Yunho Jin, Jeonghyun Choi, Sookyoung Park, Tae Ho Lee, Sang-Rae Lee, Kyu-Tae Chang, Yonggeun Hong

**Affiliations:** 1 Department of Rehabilitation Science, Graduate School of Inje University, Gimhae 50834, Korea; wy11167@naver.com (J.W.); jynh33@naver.com (Y.J.); yiopiop0011@nate.com (J.C.); 2 Ubiquitous Healthcare & Anti-aging Research Center (u-HARC), Inje University, Gimhae 50834, Korea; charm-soo@hanmail.net; 3 Biohealth Products Research Center (BPRC), Inje University, Gimhae 50834, Korea; 4 Department of Physical Therapy, College of Healthcare Medical Science & Engineering, Inje University, Gimhae 50834, Korea; 5 Division of Gerontology, Department of Medicine, Beth Israel Deaconess Medical Center, Harvard Medical School, Boston, MA 02215, USA; tlee3@bidmc.harvard.edu; 6 National Primate Research Center (NPRC), Korea Research Institute of Bioscience and Biotechnology (KRIBB), Ochang 28116, Korea; srlee@kribb.re.kr

**Keywords:** autism spectrum disorders, fragile X syndrome (FXS), sleep disorder, melatonin

## Abstract

Fragile X syndrome (FXS) is the most common monogenic form of autism spectrum disorder (ASD). FXS with ASD results from the loss of fragile X mental retardation (*fmr*) gene products, including fragile X mental retardation protein (FMRP), which triggers a variety of physiological and behavioral abnormalities. This disorder is also correlated with clock components underlying behavioral circadian rhythms and, thus, a mutation of the *fmr* gene can result in disturbed sleep patterns and altered circadian rhythms. As a result, FXS with ASD individuals may experience dysregulation of melatonin synthesis and alterations in melatonin-dependent signaling pathways that can impair vigilance, learning, and memory abilities, and may be linked to autistic behaviors such as abnormal anxiety responses. Although a wide variety of possible causes, symptoms, and clinical features of ASD have been studied, the correlation between altered circadian rhythms and FXS with ASD has yet to be extensively investigated. Recent studies have highlighted the impact of melatonin on the nervous, immune, and metabolic systems and, even though the utilization of melatonin for sleep dysfunctions in ASD has been considered in clinical research, future studies should investigate its neuroprotective role during the developmental period in individuals with ASD. Thus, the present review focuses on the regulatory circuits involved in the dysregulation of melatonin and disruptions in the circadian system in individuals with FXS with ASD. Additionally, the neuroprotective effects of melatonin intervention therapies, including improvements in neuroplasticity and physical capabilities, are discussed and the molecular mechanisms underlying this disorder are reviewed. The authors suggest that melatonin may be a useful treatment for FXS with ASD in terms of alleviating the adverse effects of variations in the circadian rhythm.

## 1. Introduction

The terms “autism spectrum disorder (ASD)” and “autism” are commonly used to describe a group of neurodevelopmental disorders that are characterized by social deficits, communication difficulties, stereotyped or repetitive behaviors, and cognitive delays. In general, individuals with autism exhibit an extensive variety of symptoms rather than identical characteristics and, thus, the newer term “ASD” has been used to describe a single diagnostic category of autism that links various conditions. ASD may be caused by several factors and, of these factors, fragile X syndrome (FXS) is thought to be the most prevalent form of the disorder [1]. This syndrome is a type of inherited intellectual disability caused by a mutation of the fragile X mental retardation 1 (*fmr1*) gene on the X chromosome. A CCG expansion repeat in the *fmr1* gene at the fragile X instability site FRAXA (Xq27.3) may result in FXS [2], and it has been reported that expanded GCC repeats in the *fmr2* gene at the FRAXE site (Xq28) can also trigger FXS but to a less severe degree than that of the *fmr1* mutation [3,4]. These mutations lead to a loss of the fragile X mental retardation protein (FMRP) which, in turn, triggers clinical abnormalities that include learning disorders, attention-deficit disorder, hyperactivity disorder, anxiety, epilepsy, sleep disturbances, and alterations in circadian behaviors [5,6,7,8]. In molecular studies, the absence of the *fmr1* gene and the fragile X-related gene 2 (*fxr2*), which is an autosomal homolog of the *fmr1* gene, alters the expression of clock gene-related components and changes the circadian rhythm [9,10]. Additionally, clinical studies have found that the sleep-related and behavioral alterations in FXS patients are associated with mutations in these two genes.

In general, sleep disorders are a problem for children with ASD and have been reported in up to 77% of children with FXS [11,12,13]. Furthermore, the occurrence of sleep disorders in individuals with FXS is associated with impaired vigilance, deficits in learning and memory, and autistic behavior with abnormal anxiety responses [14,15,16]. Children with ASD and FXS have low melatonin levels and dysregulated circadian rhythms [8,17,18,19,20]. Melatonin is an endogenous neurohormone that is predominantly synthesized in the pineal gland [21] and its major role involves regulating the circadian rhythm, which is related to the biological functions of the core body [22,23]. Many of the neurobiological effects of melatonin are mediated by melatonin receptors and involve neuronal plasticity [24,25], while melatonin receptor-independent pathways are unaffected by morphophysiological barriers, including the blood-brain barrier [26,27]. In clinical psychology fields, melatonin is commonly used to treat insomnia but has also been applied to children with autism [28]. Similarly, experimental studies have reported that melatonin treatment attenuates sleep disorders without causing side effects [29]. Thus, the present review discusses the effects of circadian dysregulation on a variety of physical and behavioral abnormalities with a focus on individuals with FXS with ASD, which is the most common monogenic type of autism and is associated with circadian dysregulation via alterations in *fmr* genes.

Oxidative stress induces brain dysfunction and increases the expression levels of oxidative biomarkers in the ASD brain [30]. For example, a number of lipofuscin-containing neurons are present in language-related cortices in ASD-affected brains as a result of oxidative stress and there are decreased levels of cellular antioxidants and altered redox metabolism in individuals with ASD [31,32,33]. Functionally, oxidative stress can produce superoxides that damage oxidative proteins and DNA; these changes are thought to contribute to the development of physiological abnormalities and psychiatric disorders in individuals with ASD. Markers of oxidative stress are associated with various neurological diseases, aging, and cases of FXS with ASD [34], and individuals with FXS with ASD exhibit higher levels of oxidative stress. Moreover, there is a close relationship between reactive oxygen species (ROS) and FMRP deficiencies [35]. In an *fmr1* knockout (KO) mouse model, which is a validated model of FXS, the antioxidant system is altered and leads to brain damage and neuronal cell death [36]. Moreover, elevated levels of intracellular ROS have been implicated in the occurrence of oxidative stress and subsequent apoptotic cell death that causes brain damage [37,38] and these oxidant factors can lead to neurotoxicity and neurodegeneration [39,40,41]. Thus, the prevention of oxidative stress by melatonin-based interventions has emerged as a novel therapeutic approach for individuals with various neurodevelopmental disorders, including autism [42,43]. Several studies have suggested that melatonin is a very powerful free radical scavenger and antioxidant [44] and recent studies have reported that melatonin has neuroprotective effects in animal models of neurological diseases [45,46,47].

FMRP deficiency may cause not only degeneration of dendrites and synapses [48], but also ROS overproduction [35]. Recently, the antioxidant effects of melatonin-induced neural plasticity have been investigated and other studies have reported the effects of melatonin on neuroplasticity and brain remodeling [49]. According to former researchers, melatonin seems to more effectively prevent lipid peroxidation in vivo [50], indicating that an even higher concentration of melatonin is thought to be required to exert its antioxidative role in vitro conditions compared to in vivo. The present review will discuss the effects of melatonin on neural regeneration and the physical capability of individuals with FXS with ASD. It is proposed here that melatonin may be a novel therapeutic candidate for FXS with ASD that may not have adverse effects resulting from variations in the circadian rhythm.

## 2. Autism Spectrum Disorder (ASD)

### 2.1. Classification of ASD

In terms of social interaction and communication, ASD is the one of the most frequently studied developmental disabilities. Children with autism commonly exhibit stereotyped behaviors within the framework of restricted and repetitive interests. Although a number of studies have attempted to elucidate the causes of autism, an exact etiology has yet to be clearly defined. Additionally, because autism is associated with several complex conditions that involve genetic predispositions and environmental triggers, a clear treatment strategy has not been suggested either. The ASD diagnosis was proposed in the fifth revision of the Diagnostic and Statistical Manual of Mental Disorders (DSM-5; American Psychiatric Association 2013 [51]) because children with autism do not show a uniform set of symptoms but, rather, a unique constellation of features particular to each individual. Therefore, the older term “autism”, which depicts a specific category of diagnoses, is being replaced by the newer term “ASD”, which better describes a postulated spectrum disorder that encompasses multiple conditions.

### 2.2. Causes of ASD

#### 2.2.1. Genetic Risk Factors

A variety of studies have consistently reported that ASD appears to be caused by hundreds of genetic variants. Thus, it is clear that there is a strong genetic risk associated with ASD and, furthermore, the genes linked with the monogenic types of ASD are involved in common signal transduction pathways related to synaptic development and neuronal plasticity. The synaptic deficits observed in ASD are induced by genetic disruptions of protein synthesis or alterations in synaptic scaffold proteins. The monogenic forms of ASD include FXS (loss of FMRP), Tuberous Sclerosis Complex (mutation of either TSC1 or TSC2), Angelman Syndrome (loss of Ube3a-dependent ubiquitination), and Phelan-McDermid syndrome (disruption of the Shank3 scaffold protein). These genetic disruptions have been utilized to develop animal models of ASD for the investigation and identification of promising candidates for ASD treatment.

#### 2.2.2. Environmental Conditions: Pre-, Peri-, and Neonatal Risk Factors of ASD

Several pre-, peri-, and neonatal complications have been identified as potential risk factors for ASD, including gestational diabetes mellitus, vaginal bleeding in the first trimester, the precipitation of medicine during pregnancy, viral and fungal infections, and meconium in the amniotic fluid. None of these factors have a conclusive cause-and-effect relationship with ASD but are more frequently present in children with ASD than in typically developing children. These types of environmental conditions can be divided into three categories of risk factors: prenatal, perinatal, and neonatal. Six prenatal factors have been consistently related to ASD: advanced maternal and paternal ages, primiparous women, bleeding, medication, and diabetes; four perinatal factors have been consistently related to ASD: induced labor, preterm birth, breech presentation, and cesarean section; and a variety of neonatal factors have been related to ASD: low birthweight and size, and poor conditions at birth including hypoxia, hyperbilirubinemia, encephalopathy, and birth defects.

## 3. Fragile X Syndrome (FXS)

### 3.1. Mechanism Underlying the Incidence of FXS

**Figure 1:**
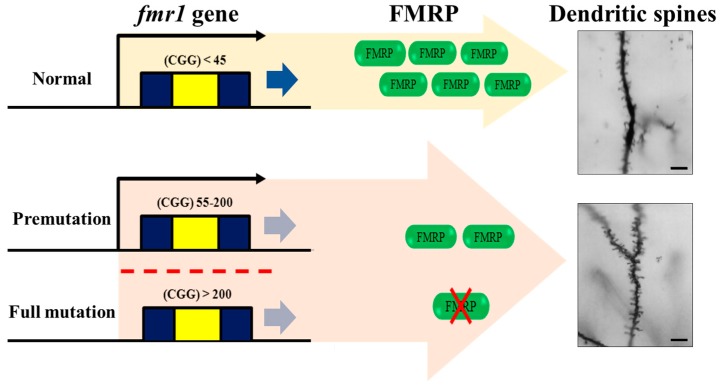
Mechanism of fragile X syndrome (FXS) incidence. Diagram of transcription and translation of the *fmr1* gene [2,54,55]. FXS resulted from the expansion of a CGG trinucleotide repeat in the 5′-untranslated region of the *fmr1* gene. Dendritic spine morphology between *fmr1* knockout (KO) and wild type mouse [56]. Overabundance of immature dendritic spine (bulbous head and a thin neck) is expressed in *fmr1* KO mouse [57,58,59]. *fmr*, fragile X mental retardation; FMRP, fragile X mental retardation protein. Scale bars = 10 µm.

FXS is the most common genetic cause of autism [52] as it affects approximately 1 in 3600 males and 1 in 4000–6000 females [53]. FXS is caused by a mutation of the *fmr1* gene at Xq27.3 on the X chromosome [2]. The mutation of the *fmr1* gene is induced by methylation at the frm1 promoter region and is associated with the expansion of the CGG triplet sequence in the 5′-untranslated region (UTR). As a result, FMRP levels are lower or absent (Figure 1). Depending on the triplet repeat mutation of the *fmr1* gene, the *fmr1* alleles are classified as normal, pre-mutation, and full mutation. In typical alleles, the *fmr1* gene contains 5–54 CGG repeats (most commonly 30 repeats) while the pre-mutation alleles range from 55 to 200 CGG repeats. Additionally, pre-mutation *fmr1* alleles are unstable and can become fully mutated alleles via maternal transmission [54,55].

### 3.2. Sleep Problems in Individuals with FXS with ASD

ASD refers to a constellation of neurodevelopmental disorders that manifest with particular behavioral characteristics. Some studies have reported that severe sleep problems are more frequent in children with autism than in typically developing children [60,61,62] and that ASD children with sleep problems tend to exhibit overactive and stereotypical behaviors. Other studies have suggested that the abnormal regulation of melatonin may be related to sleep disorders in ASD because the sleep-wake cycle is related to circadian rhythms, which are modulated by melatonin. Abnormalities in the production of melatonin might be the cause of sleep disturbances because consecutive sleep disorder has been attributed to the dysregulation of melatonin synthesis, sensitization to environmental stimuli, and behavioral insomnia syndromes. Several sleep studies have suggested that there is a correlation between sleep problems and the physiological roles of melatonin but the relationship between melatonin levels in the blood and melatonin synthesis in pinealocytes has yet to be clearly established.

### 3.3. Correlation between FXS with ASD and Circadian Rhythms

The estimated prevalence of sleep problems in individuals with FXS with ASD is approximately 80% higher than that of the general population [63] and can lead to circadian variations and altered glucose homeostasis in this population [64]. In experimental studies, mice lacking *fmr1* exhibit abnormal circadian behavioral rhythms such as a loss of rhythmic activity in a light:dark (L/D) cycle and a shorter free running period in constant darkness (DD) [9,10]. Additionally, the altered expression of the clock component has been observed in FXS animal models. The overexpression of FMRP via transfection assays increases PER1- and PER2-mediated BMAL1 (Brain muscle aryl hydrocarbon receptor nuclear translocator-like protein 1)–NPAS2 (Neuronal Per-Arnt-Sim domain protein 2) transcriptional activity [9], which suggests that FMRP is an essential component involved in the regulation of rhythmic circadian behaviors. Accordingly, *Drosophila* lacking the *fmr1* gene exhibit altered circadian rhythms [10]. Taken together, these results indicate that fragile X-related proteins might be associated with the induction of abnormal sleep patterns in FXS due to alterations in circadian genes; they may also play a critical role in the regulation of circadian output pathways.

### 3.4. Neurodevelopmental Abnormalities in FXS with ASD

Morphological analyses have consistently identified neuronal abnormalities and immature dendritic spines in most *fmr1* KO mice. Spines on neuronal dendrites with membranous protrusions have a bulbous head and a thin neck and most of these spines are associated with synaptic strength and/or the transfer of electrical signals to the axon terminal [65]. Dendritic spines release various receptor-related neurotransmitters and neurotrophic factors that enable synaptic transmission. Morphologically immature dendritic spines have been observed in *fmr1* KO mice that may lack the expression of FMRP proteins [56]. FXS patients with abnormal dendritic spine structures tend to exhibit an intellectual disability [66] and these individuals also have morphologically distinct dendritic spines such that they are longer or shorter, thinner, and fewer than those in typically developing individuals. This type of dendritic spine dysmorphogenesis is related to intellectual disabilities [67,68], and the observation of abnormal spine morphologies in FXS mice implies that their characteristics during early development can be identified [69]. In FXS, structural and functional abnormalities in dendritic spines are induced by the silencing of the *fmr1* gene, and the resultant absence of FMRP may alter the morphology and synaptic number of dendritic spines [58,70] (Figure 1). The specific role of FMRP synthesized near synapses is associated with the regulation of synaptic structure and function [59]. FMRP, which is an mRNA binding protein, stimulates the synthesis of synaptic proteins by influencing synaptic plasticity. Thus, the loss of FMRP in FXS due to *fmr1* gene silencing may imply the presence of neurodevelopmental abnormalities.

## 4. Melatonin in FXS with ASD

### 4.1. Melatonin Signaling Pathways under Normal Condition

Melatonin is a circadian synchronizer that is predominantly synthesized in the pineal gland at night. A major role of melatonin involves the regulation of biological signals associated with the L/D cycle. Many studies have demonstrated the beneficial effects of melatonin, its antioxidative and neuroprotective effects, and its involvement in neuronal plasticity and network remodeling. Melatonin synthesis begins during periods of darkness via the serotonin/*N*-acetyl serotonin (NAS)/melatonin pathway. First, the amino acid tryptophan is uptaken into the pineal gland, then tryptophan is converted into serotonin, which is converted into NAS by *N*-acetyltransferase (AANAT), and, finally, NAS is converted into melatonin by acetylserotonin *N*-methyltransferase (ASMT [71]).

### 4.2. Dysregulation of the Melatonin Pathway in FXS with ASD

**Figure 2:**
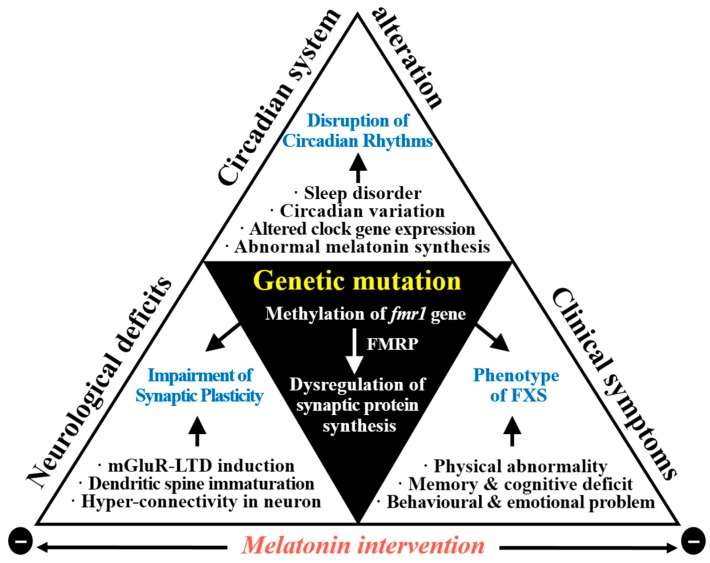
Melatonin intervention perspectives in FXS with autism spectrum disorder (ASD). Abnormal melatonin synthesis and clock-related gene mutation can result in circadian system alteration in FXS with ASD (indicated by the white upper triangular portion in the figure above). Loss of FMRP is associated with dysregulation of synaptic protein synthesis resulting in impairment of synaptic plasticity (indicated by the lower left triangular portion) and clinical symptoms (indicated by the lower right triangular portion). mGluR, metabotropic glutamate receptor; LTD, long-term depression.

Clinical studies have reported decreased levels of melatonin in the blood of individuals with FXS and ASD [19,20]. However, other studies have reported the overproduction of melatonin, which may occur to compensate pineal gland overstimulation following increased sympathetic nervous system activity [63], such as occurs with FXS [72]. Melatonin deficiencies are caused by dysfunction in its synthesis and are reflected in altered circadian rhythms (Figure 2). Sleep disturbances may be caused by significantly lower levels of melatonin as well as by significant decreases in AANAT, which is responsible for converting serotonin into NAS. Because melatonin has anxiolytic effects, ASD in conjunction with an impaired serotonin/NAS/melatonin pathway may result in circadian problems. Recent studies have reported that disruptions of the serotonin/NAS/melatonin pathway are highly sensitive and may be a useful biomarker for ASD [73,74].

### 4.3. Correlation between Melatonin with Neurodevelopmental Abnormalities in FXS with ASD

In individuals with FXS, deficits in neuronal plasticity lead to problems in learning, memory, and cognition. Recent studies have reported that FMRP modulates the number, function, and maturation of synapses and is associated with protein synthesis-dependent synaptic plasticity [71]. Because FMRP is an influential regulator of protein synthesis in dendrites, the synaptic changes associated with synaptic plasticity can be observed in FXS [75] (Figure 2). In particular, FMRP is localized in neuronal dendrites and synapses where it is thought to play a role in the regulation of local protein synthesis, such as for metabotropic glutamate receptors (mGluRs) via mRNA trafficking; thus, FMRP regulates mGluR activity as a shuttle of its mRNA [74,75]. On the other hand, decreases in FMRP expression influence long-term synaptic enhancement and are highly correlated with developmental disorders.

The activation of mGluRs induces the postsynaptic internalization of α-amino-3-hydroxy-5-methyl-4-isoxazolepropionic acid receptor (AMPA) receptors, which is modulated by the rapid translation of proteins involved in long-term depression (LTD) [76]. Lasting patterned stimuli affect neuronal synaptic plasticity achieved by both long-term potentiation (LTP) and LTD and these long-lasting enhancements tend to improve learning and memory. LTP alters synaptic connectivity by changing the morphology of postsynaptic neurons, while LTD lowers postsynaptic receptor density and leads to the elimination of old memories, which allows for the formation of new connections via the LTP process. Both of these dynamic processes are necessary to maintain the efficient development of synaptic networks and allow for the continuous receipt of new information by eliminating older less important memories. However, excess LTD proteins have been reported in mouse models of FXS, which is important because FMRP acts as an inhibitor of the translation of LTP proteins. Because the activation of mGluRs triggers the over-synthesis of LTD proteins due to a lack of FMRP, neuronal synapses with elongated and weak spine morphologies are expressed in the hippocampus and cerebellum [77]. Taken together, these findings indicate that abnormal neuronal synaptic structures negatively impact synaptic plasticity. Thus, it is possible that the inhibition of mGluRs contributes to the suppression of mGluR/LTD signaling rather than FMRP.

Experimental studies have observed excessive mGluR activation in *fmr1* KO mice [78]. Under normal conditions, FMRP is thought to be a translational repressor and to negatively regulate mGluR [79] because it is an RNA-binding protein involved in the transcriptional regulation and transport of specific mRNAs [80]. Additionally, FMRP can be highly localized in the cytoplasm of neurons and dendritic spines and acts to regulate the translation of ribosomes [81]. However, under dysfunctional FMRP conditions, mGluR activity is altered by disruptions in intracellular signaling and, subsequently, the absence of FMRP accelerates excessive mGluR5 signaling [82]. In turn, excessive mGluR5 may inhibit melatonin synthesis, as reported previously [83]. Recent studies have proposed the existence of abnormalities in melatonin secretion and circadian patterns in individuals with FXS with ASD that are likely to be due to excessive signaling via mGluRs. These receptors are a type of G-protein coupled receptor (GPCR) that can be classified into three groups (Groups I, II, and III) based on receptor structure and physiological activity [83]. mGluR Group I, which includes mGluR1 and mGluR5, is coupled to the Gq-protein subtype that activates phospholipase C [84]. mGluR Group II includes mGluR2 and mGLuR3, while Group III includes mGluRs 4, 6, 7, and 8; these groups are negatively linked with the Gi- and Go-protein subtypes, which inhibit adenylyl cyclase and suppress the formation of cyclic adenosine monophosphate (cAMP) [85].

Norepinephrine (NE)-dependent melatonin synthesis, which plays a role in the regulation of circadian rhythms and alleviates epilepsy, is suppressed by the release of glutamate [86]. mGluR Group II, particularly mGluR3, negatively regulates melatonin synthesis in pinealocytes [87] and it is known that mGluR3 and mGluR5 are expressed in pinealocytes and are involved in the negative regulation of melatonin synthesis via the inhibition of cAMP cascade. Similarly, Group II agonists suppress melatonin synthesis and prevent AANAT activity in the rat pineal gland [88]. Thus, irregularities in the synthesis of melatonin in FXS may be linked to the absence of *fmr1*, which regulates the expression of FMRP and glutamate receptors.

## 5. Melatonin as an Interventional Therapeutic Approach for FXS with ASD: Clinical Assessments

### 5.1. Effect of Melatonin as a Treatment for Sleep Disorder in FXS with ASD

Sleep disorders are common in patients with neurological diseases [89,90,91]. Additionally, the modulating properties of melatonin in terms of sleep patterns and circadian rhythms are associated with the development of ASD [92,93]. Individuals with ASD appear to be susceptible to sleep disorders and survey research has indicated that the prevalence rate of sleep issues can be as high as 89% in children with ASD and as high as 77% in children with FXS with ASD [63]. Melatonin supplements regulate the sleep-wake cycle and have been shown to alleviate sleep problems in clinical research studies. Recent studies have shown that levels of melatonin or melatonin metabolites are significantly lower in children with ASD than in typically developing children [17,19]. Melatonin is an endogenous neurohormone produced in pinealocytes, which are neuroendocrine cells, and is widely used in clinics to treat insomnia in children because it is inexpensive, efficient for treating sleep problems, and has no side effects [94]. For example, a study investigating the efficacy of melatonin in 107 children with autism (age range: 2–18 years old) found that 25% of the treated children no longer reported sleep problems and that 60% of the treated children reported improvements in sleep [95]. It has also been shown that autistic children have lower melatonin levels than typically developing children [96].

### 5.2. Effects of Melatonin on Cognitive and Learning Disabilities in FXS with ASD

Chronic sleep disorders are typically associated with learning and behavior issues in ASD individuals, and the functional consequences of abnormal melatonin levels in individuals with FXS may also include learning and memory problems. In *fmr1* KO mice, abnormality of dendritic spines can be seen [97], which is known to be harmful to memory function [98]. However, several studies have reported that melatonin facilitates synaptic plasticity and enhances the mechanisms underlying learning and memory [24,99,100]. Taken together, these findings suggest that there is a correlation between the loss of neuroplasticity and the malfunctioning of, or irregularities in, melatonin production in FXS.

Low melatonin levels are associated with altered activity in the GABAergic system [13]. γ-aminobutyric acid (GABA) is the main inhibitory neurotransmitter in the central nervous system (CNS) and, accordingly, induces the relaxation of the brain and sleep. Melatonin stimulates GABAergic activity in the brain and, thus, abnormal melatonin levels influence the onset and length of sleep [101]. Similarly, alterations in the circadian clock mechanism due to abnormal melatonin synthesis can affect the sleep-wake cycle. Recently, studies using animal models of autism have indicated that clock and clock-related genes may interact in the ASD phenotype and studies using *fmr1* KO mice have implicated clock proteins in sleep alterations in FXS. Furthermore, melatonin is helpful for treating sleep problems in cases of autism with oxidative stress and the physical alterations of axons and dendritic spines [92,102].

### 5.3. Neuroprotective Effects of Melatonin on Seizures in FXS with ASD

Individuals with FXS have a higher risk of neurological diseases, such as seizures, which are an important characteristic of autism and significantly associated with FXS. Clinical survey data have revealed that epilepsy occurs in 10–20% of children with FXS [103]. However, a recent study found that melatonin, which is used to treat sleep disorders and does not cause side effects, can effectively regulate severe epilepsy [104] as well as suppress its incidence.

Epilepsy is a neurological disease that is accompanied by biochemical responses resulting from brain injuries and chemical imbalances. In particular, free radicals are linked to seizure initiation [105]. The excessive production of free radicals contributes to brain damage in patients with neuropathological conditions such as stroke, Alzheimer’s disease, and Parkinson’s disease [106]. Oxidative stress occurs in mitochondria following seizures, which constitute a primary cause of oxidative stress that is critical to neuronal cell fates. Recently, melatonin was discussed in relation to epileptic seizures [107,108,109] because this neurohormone is known to act as an antioxidant and free radical scavenger. A large study reported that melatonin may be a promising anticonvulsant candidate due to its capability to induce antiepileptic activity, and a clinical study showed that low baseline levels of melatonin are observed in patients who suffer from epilepsy but that these levels dramatically increase after a seizure. Although these findings indicate that melatonin may play a role in the regulation of seizures, other studies have reported that melatonin might actually increase the risk of seizure. The latter findings are supported by evidence showing that melatonin may affect hippocampal excitability and can increase one’s susceptibility for seizures by lowering the seizure threshold [110]. Thus, the role of melatonin in epilepsy remains under debate.

### 5.4. Synergistic Effects of Melatonin on Synaptic Plasticity in FXS with ASD

Recently, it was reported that melatonin acts as a neuroprotective agent against neurological injuries [111,112,113,114]. The neuroprotective effects of melatonin have been demonstrated by in vivo studies that co-administered injections of melatonin and dexamethasone, the latter of which is known to be neurotoxic to cells in the hippocampus. The animals that received these injections exhibited decreased numbers of abnormal hippocampal cells and different histological properties compared to the vehicle group. Melatonin is an endogenous neurohormone that regulates several biological functions but exogenous melatonin has also been shown to prevent neuronal cell death and improve cognitive dysfunction [115]. The neuroprotective effects of melatonin have also been demonstrated in cases of acute global cerebral ischemia and hypoxic ischemia [116]. Most cerebral ischemic models produce a significant loss of neurons in the hippocampal CA1 to CA4 regions. In the case of children who experienced perinatal hypoxic ischemia, the resulting brain injuries were directly caused by neuronal cell death and then indirectly by chronic neuropathic conditions such as cerebral palsy, intellectual disabilities, and epilepsy [117]. On the other hand, brain damage due to cerebral ischemia is primarily induced by reductions in the blood supply and treatment may include oxygen supplementation. When the blood supply becomes blocked, the cellular metabolic system converts to anaerobic metabolism and, consequently, depletions in adenosine triphosphate (ATP), the accumulation of lactic acid, and the cellular input of calcium may occur.

However, melatonin treatment can protect the brain from damage and delay neuronal cell death. During reperfusion after cerebral artery occlusion, the overproduction of free radicals triggers the activation of oxidative stress but melatonin may suppress this type of brain damage. Because melatonin contributes to various forms of neuroplasticity, including learning, memory, and recovery from brain damage, the manner in which this hormone acts as a neuroplastic agent after autism should be elucidated. Interestingly, the delayed stabilization and abnormal morphological features of dendritic spines are the main characteristics of FXS, and these are related to impaired synaptic signaling and connections. Disruptions in the pruning of excitatory synapses and hyperconnectivity have been observed in FXS patients and the *fmr1* KO mouse model due to loss of postsynaptic FMRP [118]. Deficits in FMRP in conjunction with incomplete pruning induces cell-to-cell hyperconnections in synapses. Moreover, the connection pruning process that occurs during early development is essential for the formation of normal neuronal circuits. In contrast, in FXS, FMRP dysfunction results in hyperconnectivity and an excessive number of synapses that lead to autistic features. Melatonin has been associated with neurogenesis and microtubule polymerization in dendrites and, thus, melatonin may stimulate dendrite maturation and affect neuroregeneration.

## 6. Conclusions

Molecular biological research on autism has produced a significant number of therapeutic candidates, including melatonin. The present review aimed to highlight the neurological effects of melatonin in FXS with ASD, and investigate prevention strategies as well as therapeutic approaches for the management of FXS. The clinical application of melatonin-based therapies is expected to have high efficacy and to suppress the onset of diseases. However, additional studies should be conducted to determine the mechanisms underlying the beneficial effects of melatonin on autism and FXS.
